# The PTS transporters of *Lactobacillus gasseri *ATCC 33323

**DOI:** 10.1186/1471-2180-10-77

**Published:** 2010-03-12

**Authors:** Alyssa L Francl, Taksawan Thongaram, Michael J Miller

**Affiliations:** 1 Department of Food Science and Human Nutrition, University of Illinois, 905 S. Goodwin Ave., Urbana, IL, USA; 2 Division of Nutritional Sciences, University of Illinois, 905 S. Goodwin Ave., Urbana, IL, USA; 3 Danisco, USA, Inc., 3329 Agriculture Drive, Madison, WI 53716, USA

## Abstract

**Background:**

Lactobacilli can utilize a variety of carbohydrates which reflects the nutrient availability in their respective environments. A common lactobacilli in the human gastrointestinal tract, *Lactobacillus gasseri*, was selected for further study. The currently available annotation of the *L. gasseri *ATCC 33323 genome describes numerous putative genes involved in carbohydrate utilization, yet the specific functions of many of these genes remain unknown.

**Results:**

An enzyme I (*EI*) knockout strain revealed that a functional phosphotransferase transporter system (PTS) is required to ferment at least 15 carbohydrates. Analysis of the *L. gasseri *ATCC 33323 genome identified fifteen complete (containing all of the necessary subunits) PTS transporters. Transcript expression profiles in response to various carbohydrates (glucose, mannose, fructose, sucrose and cellobiose) were analyzed for the fifteen complete PTS transporters in *L. gasseri*. PTS 20 was induced 27 fold in the presence of sucrose and PTS 15 was induced 139 fold in the presence of cellobiose. No PTS transporter was induced by glucose, fructose or mannose. Insertional inactivation of PTS 15 and PTS 20 significantly impaired growth on cellobiose and sucrose, respectively. As predicted by bioinformatics, insertional inactivation of PTS 21 confirmed its role in mannose utilization.

**Conclusions:**

The experiments revealed the extensive contribution of PTS transporters to carbohydrate utilization by *L. gasseri *ATCC 33323 and the general inadequacy of the annotated sugar specificity of lactobacilli PTS transporters.

## Background

Lactic Acid Bacteria (LAB) are a group of functionally and genetically related bacteria known for the fermentation of sugars to the metabolic end-product, lactic acid [1]. LAB belong to the order of *Lactobacillales*, which includes the genera *Lactobacillus*, *Lactococcus*, *Leuconostoc*, *Oenococcus*, *Pediococcus*, *Streptococcus*, among others [2]. LAB, including lactobacilli, are very diverse and are commonly found in many different environments. Lactobacilli are naturally associated with many foods, including fruits, vegetables, cereal grains, wine, milk and meats. In addition, several species of *Lactobacillus*, such as *Lactobacillus gasseri*, are considered to be indigenous to the gastrointestinal tract (GIT) and other mucosal surfaces, including the mouth and vagina [3,4]. The *Lactobacillus *genus has been explored for their probiotic potential due to the ability of specific strains to survive passage through the human GIT and exert benefits to general health and wellness to the host [5]. Probiotics have been defined as live microorganisms that, when administered in adequate amounts, confer a health benefit to the host [6]. Some of these benefits include a positive influence on the normal microbiota present in the GIT, the competitive exclusion of pathogens, and the stimulation or adjustment of mucosal immunity [7].

Lactobacilli can utilize a variety of carbohydrates which reflects the nutrient availability in their respective environments. In many lactobacilli, PTS (phosphotransferase system) transporters are the dominant carbohydrate transporters [8]. For example, the *L. plantarum *genome revealed 25 PTS transporters which correlate with its broad carbohydrate utilization profile [9]. Analysis of the *L. johnsonii*, *L. acidophilus *and *L. gasseri *genomes further substantiate these observations since they contain a preponderance of PTS transporters [10]. The PTS functions by the transfer of a phosphate group from phosphoenolpyruvate (PEP) to the incoming sugar through a series of sequential steps that involve the different components of the PTS. The PTS consists of cytoplasmic components, which lack sugar specificity, and membrane-associated enzymes, which are specific for a few sugars, at most. The cytoplasmic components are enzyme I (*EI*) and histidine-phosphorylatable protein (HPr). The membranous component of the PTS system, enzyme II (EII), is made up of three to four subunits: IIA, IIB, IIC and sometimes IID [11].

In reference to the human GIT, lactobacilli are the predominant species in the ileum [12]. The carbohydrate utilization profile of lactobacilli isolated from porcine ileal contents reflects the carbohydrate content of the diet [13]. For example, the relative percentage of lactobacilli that can utilize starch increases after weaning, whereas the relative percentage of lactobacilli that can utilize lactose decreases after weaning. Carbohydrate transporters, including PTS transporters, are among the genes that have been shown to be expressed during GIT transit of lactobacilli [14,15]. The importance of PTS transporters in *Lactobacillus johnsonii *NCC 533 has been verified by studying gut persistence *in vivo*. Specifically, expression of a PTS transporter annotated as mannose-specific is required for the long-residence phenotype of *L. johnsonii *NCC 533 [15].

Genome sequencing of selected lactobacilli has enabled researchers to make additional conclusions about the traits and characteristics of these organisms. In 2006, the sequenced genomes of *L. gasseri *ATCC 33323 and many other lactobacilli were released [16]. The currently available annotation of the *L. gasseri *ATCC 33323 genome describes numerous genes potentially involved in the uptake and metabolism of carbohydrates, yet the specific functions of these genes remain unknown. Our objective was to characterize PTS transporter functionality in *L. gasseri *ATCC 33323 using gene knockouts, bioinformatics, comparative carbohydrate utilization assays and transcript expression profiles.

## Results and Discussion

### Identification of PTS-Transported Carbohydrates

As the most common method of carbohydrate utilization in some lactobacilli [17], the PTS transporters in *L. gasseri *ATCC 33323 were selected for further study. PTS transporters require a functional *EI *to import carbohydrates [18]. Additionally, some non-PTS carbohydrate transporters also require a functional PTS system for full transport activity [19,20]. Insertional inactivation of *EI *in *L. gasseri *was performed to identify the carbohydrates which require a functional PTS system for utilization (Table 1). *L. gasseri *ATCC 33323 *EI *was only able to utilize 2 (D-glucose and D-maltose) of the 17 carbohydrates that the parent strain could utilize, indicating that transporters independent of the PTS system can import these two carbohydrates. The 15 carbohydrates that can be utilized by *L. gasseri *ATCC 33323 but not by *L. gasseri *ATCC 33323 *EI *are D-galactose, D-fructose, D-mannose, N-acetylglucosamine, amygdalin, arbutin, esculin ferric citrate, salicin, D-cellobiose, D-lactose, D-saccharose (sucrose), D-trehalose, amidon (starch), gentiobiose and D-tagatose. These 15 carbohydrates are either (1) imported directly by a PTS transporter and/or (2) imported by a non-PTS carbohydrate transporter that requires a functional PTS system. Examples of non-PTS transporters that require a functional PTS system to import sugars include LacS [19] and RafP [20]. Both LacS and RafP have a PTS IIA-glc domain (PF00358) fused to a permease domain. The PTS IIA-glc domain of these proteins is required for full transport activity. All PTS IIA domains identified in the Conserved Domain Database [21] for *L. gasseri *ATCC 33323 are a part of PTS transporters. Additionally, *L. gasseri *ATCC 33323 does not have homologs to LacS or RafP. Consequently, we can confirm that (1) *L. gasseri *ATCC 33323 does not have a LacS or RafP, and (2) *L. gasseri *ATCC 33323 does not have a PTS IIA domain fused to a non-PTS transporter. While it is still possible that there are unknown PTS IIA domains that have not been characterized, we conclude that the majority of these 15 carbohydrates are imported by PTS transporters.

**Table 1:** Carbohydrate utilization profiles of various lactobacilli

Carbohydrate	*L. gasseri *ATCC 33323 ^a^	*L. gasseri *ATCC 33323 *EI*::MJM75	*L. gasseri *ADH	*L. gasseri *ATCC 19992
D-galactose	+	-	+	+
D-glucose	+	+	+	+
D-fructose	+	-	+	+
D-mannose	+	-	+	+
N-acetylglucosamine	+	-	+	+
Amygdalin	+	-	-	-
Arbutin	+	-	-	-
Esculin ferric citrate	+	-	+	+
Salicin	+	-	-	-
D-cellobiose	+	-	+	+
D-maltose	+	+	+	+
D-lactose (bovine origin)	+	-	+	+
D-saccharose (sucrose)	+	-	+	+
D-trehalose	+	-	+	+
Amidon (starch)	+	-	+	-
Gentiobiose	+	-	+	+
D-tagatose	+	-	+	+

The carbohydrate utilization profiles of *L. gasseri *ATCC 33323, *L. gasseri *ATCC 33323 *EI*::MJM75, *L. gasseri *ADH and *L. gasseri *ATCC 19992 were determined using API 50 CH assays after 48 hours incubation. The ability or inability to utilize carbohydrates is represented by "+" or "-", respectively. The superscript indicates the following: a -- there were no differences among the carbohydrate utilization profiles of *L. gasseri *ATCC 33323 *PTS 15*::MJM99, *L. gasseri *ATCC 33323 *PTS 20*::MJM100, *L. gasseri *ATCC 33323 *PTS 21*::MJM101 and *L. gasseri *ATCC 33323.

PTS transporters with specificities for many of these carbohydrates (arbutin, amygdalin, salicin, gentiobiose and tagatose) have not been identified amongst lactobacilli. For several of the other carbohydrates, very few PTS transporters have been identified amongst lactobacilli. For example, PTS transporters for D-galactose and D-lactose have only been identified in *L. casei *[22,23], whereas many other lactobacilli utilize permeases [24,20]. Carbohydrates that can be utilized by both *L. gasseri *ATCC 33323 and *L. gasseri *ATCC 33323 *EI *(D-glucose and D-maltose) can be transported into the cell by non-PTS mechanism(s). The *L. gasseri *genome encodes two putative permeases with a predicted specificity for glucose [3]. A putative sugar ABC transporter has also been predicted for maltose [3]. The importance of PTS transporters in *L. gasseri *ATCC 33323 was revealed based on the carbohydrate utilization profiles of the wild type and *EI *knockout strains.

### PTS Transporters in Lactobacilli

Bioinformatic analysis was used to characterize the PTS transporters of the sequenced lactobacilli genomes. In total, eleven different species were analyzed, including *Lactobacillus acidophilus *NCFM, *L. brevis *ATCC 367, *L. casei *ATCC 334, *L. delbrueckii ssp. bulgaricus *ATCC 11842, *L. delbrueckii ssp. bulgaricus *ATCC BAA-365, *L. gasseri *ATCC 33323, *L. johnsonii *NCC 533, *L. plantarum *WCFS1, *L. reuteri *F275, *L. sakei ssp. sakei *23 K and *L. salivarius ssp. salivarius *UCC118. A complete PTS transporter was defined as having the IIA, IIB and IIC components present in the enzyme II of the PTS. An incomplete PTS lacked one or more of these three subunits [25].

Table 2 lists the eleven different lactobacilli and the number of complete and incomplete PTS(s) found in each organism. The number of PTS transporters in the selected lactobacilli analyzed varies greatly. *L. plantarum *WCFS1 has the most complete PTS transporters with 25, whereas *L. reuteri *F275 and *L. brevis *ATCC 367 have no complete PTS transporters. The closely related *L. gasseri *ATCC 33323, *L. johnsonii *NCC 533 and *L. acidophilus *NCFM had 15, 16 and 10 complete PTS transporters, respectively.

**Table 2:** Complete and incomplete PTS transporters in selected lactobacilli

Organism	Complete PTS	Incomplete PTS
*L. acidophilus *NCFM	10	13
*L. brevis *ATCC 367	0	5
*L. casei *ATCC 334	17	14
*L. delbrueckii ssp. bulgaricus *ATCC 11842	2	7
*L. delbrueckii ssp. bulgaricus *ATCC BAA-365	2	4
*L. gasseri *ATCC 33323	15	10
*L. johnsonii *NCC 533	16	9
*L. plantarum *WCFS1	25	13
*L. reuteri *F275	0	4
*L. sakei ssp. sakei *23 K	5	6
*L. salivarius ssp. Salivarius *UCC118	7	3

Complete transporters were defined as having the IIA, IIB and IIC subunits of EII present, and incomplete transporters were defined as lacking at least one subunit.

The number of PTS transporters present in a species has been proposed to be due to the adaptation of species to their specific niches [26]. Species such as *L. gasseri *ATCC 33323, *L. acidophilus *NCFM and *L. johnsonii *NCC 533 all have more PTS transporters than most of the other species. These common inhabitants of the GIT may require a large number of PTS transporters to survive in their environment. *L. delbrueckii *species are commonly used in dairy fermentations, where the nutrient-rich environment has less carbohydrate diversity and has resulted in significant gene loss in respect to carbohydrate utilization [27].

In an effort to characterize PTS transporters through bioinformatics, seven different PTS families have been differentiated [25] and are available at the Transport Classification Database [28]. Table 3 lists the PTS transporter families for all of the complete and incomplete PTS transporters in *L. gasseri *ATCC 33323. Two of the three complete PTS transporters from the LAC family (PTS 6 and 9) have no known homologs amongst the 10 other lactobacilli analyzed (listed in Table 2). In addition, PTS 8, of which none of the other 10 analyzed lactobacilli have a complete homolog, is the only complete PTS member of the GAT family in *L. gasseri *ATCC 33323. There are no members of the GUT and ASC family amongst the 15 complete PTS transporters of *L. gasseri *ATCC 33323.

**Table 3:** Current annotations and predicted substrates of the PTS transporters in *L. gasseri *ATCC 33323

PTS	ORF	Current annotation	Predicted Function	**TCDB Family **[40]
1B	117	PTS, mannose/fructose/N-acetylgalactosamine-specific component IIB		4.A.6
1C	118	PTS, mannose/fructose/N-acetylgalactosamine-specific component IIC		
1D	119	PTS, mannose/fructose/N-acetylgalactosamine-specific component IID		
1A	120	PTS, mannose/fructose-specific component IIA		

2A	125	Phosphotransferase system galacitol-specific IIA domain (Ntr-type)		4.A.6

3BCA	149	PTS fructose-specific component IIB		4.A.2

4C	187	Cellobiose-specific PTS system IIC component		4.A.3

5A	192	Cellobiose-specific PTS system IIA component		4.A.3
5B	194	Cellobiose-specific PTS system IIB component		
5C	195	Cellobiose-specific PTS system IIC component		

6A	342	Cellobiose-specific PTS system IIA component	Lactose ^b,c,d^; Galactose ^c^	4.A.3
6CB	343	Cellobiose-specific PTS system IIC component		

7BCA	398	Sucrose PTS, EIIBCA		4.A.1

8A	495	PTS, galacitol-specific IIA domain (Ntr-type)	Lactose ^c^; Galactose ^c^	4.A.5
8B	496	PTS, galacitol-specific IIB component		
8C	497	Galactitol PTS, EIIC		

9A	500	Cellobiose-specific PTS system IIA component		4.A.3
9CB	501	Cellobiose-specific PTS system IIC component		

10B	514	PTS, mannose/fructose/N-acetylgalactosamine-specific component IIB	Galactose ^c^	4.A.6
10C	515	PTS, mannose/fructose/N-acetylgalactosamine-specific component IIC		
10D	516	PTS, mannose/fructose/N-acetylgalactosamine-specific component IID		
10A	517	PTS, mannose/fructose-specific component IIA		

11ABC	535	Beta-glucoside-specific PTS system IIABC component	Trehalose ^a^	4.A.1

12C	570	Cellobiose-specific PTS system IIC component		4.A.3

13A	1348	Glucitol/sorbitol PTS, EIIA		--

14C	1430	Cellobiose-specific PTS system IIC component		4.A.3

15BCA	1669	Trehalose PTS trehalose component IIBC	Cellobiose ^c,d^; β-glucosides ^a^; Galactose ^c^	4.A.1

16C	1676	Cellobiose-specific PTS system IIC component		4.A.3

17CBA	1688	N-acetylglucosamine and glucose PTS, EIICBA		4.A.1

18ABC	1726	Fusion of IIA, IIB and IIC component of mannitol/fructose-specific PTS	Fructose ^b^	4.A.2

19BCA	1755	Beta-glucosides PTS, EIIBCA		4.A.1

20BCA	1778	Sucrose PTS, EIIBCA	Sucrose ^b,c,d^	4.A.1

21D	1793	Mannose-specific PTS system component IID	Glucose ^a^; Mannose ^a,d^	4.A.6
21C	1794	Mannose-specific PTS system component IIC		
21AB	1795	PTS, mannose/fructose-specific component IIAB		

22C	1811	Cellobiose-specific PTS system IIC component		4.A.3

23C	1835	Galacitol PTS, EIIC		4.A.5

24C	1836	Galacitol PTS, EIIC		4.A.5

25C	1851	Cellobiose-specific PTS system IIC component		4.A.3

The superscripts for the predicted functions indicate the following: a -- homology to characterized PTS transporters in other species; b -- homology to PTS transporters that are induced by a particular carbohydrate(s) in other species; c -- PTS transporters that are induced by a particular carbohydrate in *L. gasseri *ATCC 33323; and d -- characterization in *L. gasseri *ATCC 33323. The TCDB family names are categorized as follows: 4.A.1 -- PTS glucose-glucoside (GLC); 4.A.2 -- PTS fructose-mannitol (FRU); 4.A.3 -- PTS Lactose-N,N'-Diacetylchitobiose-β-glucoside (LAC); 4.A.5 -- PTS Galactitol (GAT); and 4.A.6 -- PTS Mannose-Fructose-Sorbose (MAN) [40].

### Strain Variation

In order to determine the variability of PTS transporters within *L. gasseri*, fifteen complete PTS transporters in *L. gasseri *ATCC 33323 were compared to *L. gasseri *ADH and *L. gasseri *ATCC 19992 using PCR (Table 4). PCR products were obtained for all of the fifteen PTS transporters when *L. gasseri *ATCC 33323 was used as the template. There was no visible amplicon for PTS 6 and 9 for either *L. gasseri *ADH or ATCC 19992. In addition, there was no visible amplicon for PTS 7 and 10 in *L. gasseri *ADH. The PCR of all other PTS transporters resulted in a visible product for *L. gasseri *ADH and *L. gasseri *ATCC 19992. The PTS transporters that are unique to *L. gasseri *ATCC 33323 amongst sequenced lactobacilli (PTS 6, 7 and 9) also appear to be variable within *L. gasseri*.

**Table 4:** Presence of complete *L. gasseri *ATCC 33323 PTS transporters in other *L. gasseri *strains

*L. gasseri *ATCC 33323 PTS	*L. gasseri *ATCC 33323	*L. gasseri *ADH	*L. gasseri *ATCC 19992
1	+	+	+
3	+	+	+
5	+	+	+
6	+	-	-
7	+	-	+
8	+	+	+
9	+	-	-
10	+	-	+
11	+	+	+
15	+	+	+
17	+	+	+
18	+	+	+
19	+	+	+
20	+	+	+
21	+	+	+

The presence or absence of a visible PCR gel product in *L. gasseri *ATCC 33323, *L. gasseri *ADH and *L. gasseri *ATCC 19992 is denoted by "+" or "-", respectively.

Recently, draft genomic DNA sequences have become publicly available from three *L. gasseri *strains (202-4, MV-22 and JV-V03). Bioinformatic analysis of the *L. gasseri *draft genomes revealed that PTS 7, 10 and 15 from *L. gasseri *ATCC 33323 are not present in all *L. gasseri *strains whereas the other 12 complete PTS transporters in *L. gasseri *ATCC 33323 where also found in *L. gasseri *202-4, *L. gasseri *MV-22 and *L. gasseri *JV-V03. While caution should be used to interpret the draft genomes since they are unfinished, it is interesting to note that PTS 7 and PTS 10 were found to be variable amongst *L. gasseri *using both PCR and bioinformatic approaches.

Carbohydrate utilization assays were also used to study different *L. gasseri *strains in comparison to *L. gasseri *ATCC 33323. *L. gasseri *ADH and *L. gasseri *ATCC 19992 had different carbohydrate utilization profiles when compared to *L. gasseri *ATCC 33323, as shown in Table 1. Among the *L. gasseri *strains, only *L. gasseri *ATCC 33323 was able to grow on amygdalin, arbutin and salicin. Both *L. gasseri *ATCC 33323 and *L. gasseri *ADH were able to grow on amidon (starch), but *L. gasseri *ATCC 19992 was not able to grow on amidon. Also, there were no carbohydrates that were unique to *L. gasseri *ATCC 19992. As previously indicated [29], these results demonstrate the potential for gain/loss of carbohydrate utilization genes which results in difficulty in using carbohydrate utilization assays for species identification.

### Transcript Expression Profiles

**Figure 1:**
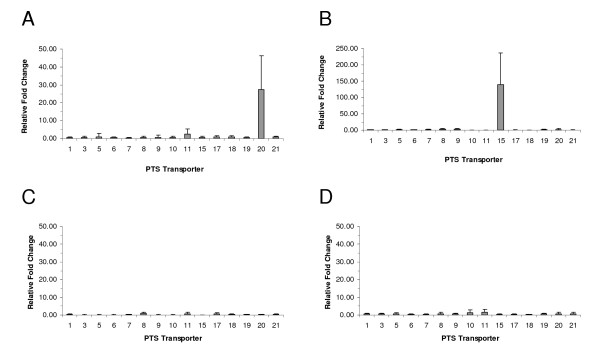
**Relative fold changes of the complete PTS transporters in *L. gasseri *ATCC 33323**. Cells grown in semi-synthetic MRS + selected carbohydrate were compared to cells grown in semi-synthetic MRS + fructose. Selected carbohydrates were sucrose (A), cellobiose (B), glucose (C) and mannose (D). RNA was extracted from log phase cells and subjected to two-step real-time PCR. Results are the average of three independent experiments, and error bars indicate standard deviations.

Real-time PCR was used to study the transcript expression profiles of the fifteen complete PTS transporters in *L. gasseri *ATCC 33323 in response to fructose (calibrator), glucose, mannose, cellobiose and sucrose. PTS 7 and PTS 20 were annotated as being sucrose-specific and both have adjacent ORFs annotated at sucrose-6-phosphate hyrdolase. PTS 20 was induced 27 ± 19 fold with sucrose as the sole carbohydrate source, whereas all other PTS transporters were induced less than 3 fold (Figure 1A). The *L. acidophilus *NCFM PTS transporter (ORF 401) induced by sucrose [24] is a homolog of PTS 20 (80% amino acid identity). In fact, *L. johnsonii *NCC 533 ORF 519 is also a homolog to PTS 20 in *L. gasseri *(98% amino acid identity), and all three strains can utilize sucrose.

In the presence of cellobiose, PTS 15 was induced 139 ± 97 fold (Figure 1B). All other PTS transporters were induced less than 5 fold. *L. acidophilus *NCFM has a homolog to PTS 15 (ORF 725 at 62% amino acid identity) and is able to utilize cellobiose. Surprisingly, three of the complete PTS transporters of *L. gasseri *ATCC 33323 were annotated as cellobiose-specific (PTS 5, 6 and 9), yet none demonstrated inducible expression in the presence of cellobiose. The annotation of PTS 15 incorrectly indicates a specificity for trehalose, yet PTS 11 is a homolog for the characterized trehalose PTS in *L. acidophilus *NCFM [30]. Our results demonstrate the importance of determining PTS transcript expression profiles to identify PTS transporter specificity rather than relying solely on annotation and bioinformatics.

There were no PTS transporters that were significantly induced in the presence of glucose or mannose (Figures 1C and 1D, respectively). The PTS transporter for glucose is constitutively expressed in *Streptococcus mutans *[31], *S. bovis *[32], and *Lactobacillus casei *[33]. Additionally, no PTS transporter was induced by glucose in *L. acidophilus *NCFM [24]. PTS 21 includes a fused IIA and IIB domain (ORF 1795), in addition to the enzyme IID (ORF 1793) subunit which are characteristic of glucose PTS transporters [34]. In addition, PTS 21 is a homolog of the characterized glucose/mannose PTS transporter in *L. casei *[33], providing evidence that PTS 21 may be involved in the transport of glucose. Homologs of PTS 21 are found in all 8 of the sequenced lactobacilli genomes we analyzed that contain at least one complete PTS transporter. *L. gasseri *ATCC 33323 *EI *indicates that a non-PTS mechanism is able to import glucose as well (Table 1). While no PTS transporter was induced by mannose (Figure 1D), PTS transporter function is required for the utilization of mannose (Table 1), suggesting that the glucose permease(s) is unable to transport mannose. Since the glucose PTS transporter can also transport mannose in some instances [31], and that the PTS 21 homolog in *L. casei *ATCC 393 transports glucose and mannose [33,35], we predict that PTS 21 also transports glucose and mannose.

### Insertional Inactivation of PTS 15, PTS 20 and PTS 21

In order to confirm the conclusions from bioinformatic and transcript analyses, gene knockouts for PTS 15 (MJM99), PTS 20 (MJM100) and PTS 21 (MJM101) were created. Carbohydrate utilization assays were used to characterize MJM99, MJM100 and MJM101 (Table 1). No differences were detected among these three knockout strains and the parental strain. The qualitative nature of the carbohydrate utilization assay prevented the ability to characterize these knockout strains.

**Figure 2:**
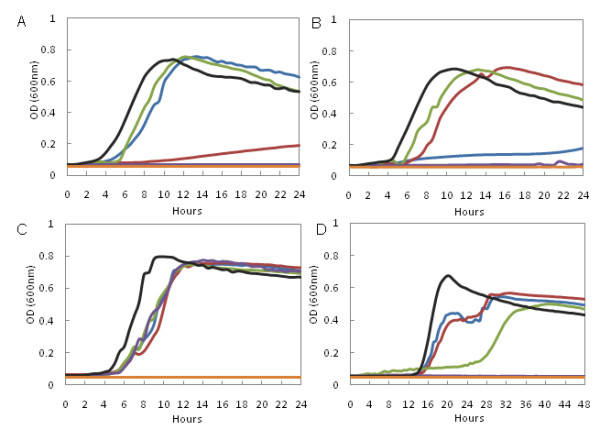
**Growth curves of selected L. gasseri strains**. Growth curves of MJM99 (blue), MJM100 (red), MJM101 (green), MJM75 (purple), NCK334 (black) and an uninoculated control (orange) grown in semi-synthetic MRS + selected carbohydrate. Selected carbohydrates were sucrose (A), cellobiose (B), glucose (C) and mannose (D). Results are the average of duplicate wells from one of three independent experiments.

Growth curves were performed with MJM99, MJM100, MJM101, *L. gasseri *ATCC 33323 (NCK334) and *L. gasseri *ATCC 33323 *EI *(MJM75) (Figure 2). The growth media had sucrose (Figure 2A), cellobiose (Figure 2B), glucose (Figure 2C) or mannose (Figure 2D) as the sole carbohydrate. In all four cases, *L. gasseri *ATCC 33323 *EI *did not grow and was indistinguishable from the non-inoculated control. Growth of MJM100 was significantly reduced on sucrose (Figure 2A), confirming the bioinformatic and transcript expression profile based prediction. Growth of MJM99 was significantly reduced on cellobiose (Figure 2B), confirming the transcript expression profile based prediction. In regards to glucose, the growth of all four knockout strains was similar to the parental strain (Figure 2C). MJM101 had a significantly extended lag phase that was approximately 10 hours longer than the lag phase observed with the other analyzed strains when mannose was the sole carbohydrate (Figure 2D). PTS 21 and another unidentified PTS transporter(s) import mannose.

### Prediction of *L. gasseri *ATCC 33323 PTS Transporter Specificities

We have identified 15 carbohydrates that require a functional PTS system for utilization (Table 1): galactose, fructose, mannose, N-acetylglucosamine, amygdalin, arbutin, esculin ferric citrate, salicin, cellobiose, lactose, sucrose, trehalose, starch, gentiobiose and tagatose. The annotations of the complete and incomplete PTS transporters are presented in Table 3. Sucrose induced expression of PTS 20 (Figure 1A), and cellobiose induced expression of PTS 15 (Figure 1B). Insertional inactivation of PTS 20 and PTS 15 significantly reduced growth on sucrose (Figure 2B) and cellobiose (Figure 2C), respectively. Based on transcription expression profiles, bioinformatics and the characterization of a PTS 21 knockout strain, we predict that PTS 21 can transport glucose and mannose [33].

We could not detect increased PTS expression from any of the 15 complete PTS transporters when grown in fructose with the glucose condition as the calibrator. However, PTS 3 and PTS 18 are two candidates for fructose transport. Both PTS 3 and PTS 18 co-localize with ORFs (LGAS_0148 and LGAS_1727, respectively) which have a fructose-1-phosphate kinase domain (FruK; COG 1105). PTS 18 is a homolog to the PTS transporter in *L. acidophilus *(LBA1777) which is induced in the presence of fructose [24], yet we were unable to demonstrate induction of PTS 18 or any other complete PTS transporter with fructose. PTS 3 does not have a homolog in *L. acidophilus *NCFM. Additionally, PTS 3 and/or PTS 18 may be involved in tagatose utilization. The potential activity of COG 1105 includes tagatose-6-phosphate kinase which is required for the tagatose-6-phosphate pathway. Unfortunately, no PTS transporter amongst LAB has been demonstrated to transport tagatose. However, *L. acidophilus *NCFM is unable to utilize tagatose and also lacks a homolog for PTS 3. Functional characterization is required to determine if PTS 3 and/or PTS 18 transports fructose and/or tagatose.

Previous studies have identified a lactose permease in the closely related *L. acidophilus *NCFM [24]. However, *L. gasseri *ATCC 33323 does not have a homolog for the lactose permease from *L. acidophilus *NCFM. Rather, *L. gasseri *ATCC 33323 uses PTS transporters to import lactose. PTS 6 and PTS 8 are induced by lactose [36]. Analysis of *L. gasseri *PTS 6, *L. gasseri *PTS 8 and *L. gasseri *PTS 6 PTS 8 revealed that PTS 6 is required for maximum fermentation of lactose [36]. The only lactose PTS transporter previously characterized in lactobacilli has been with *L. casei *[22,23]. Galactose induced several PTS transporters (PTS 6, 8, 10 and 15) [36]. Similar to lactose, analysis of *L. gasseri *PTS 6, *L. gasseri *PTS 8 and *L. gasseri *PTS 6 PTS 8 revealed that PTS 6 is required for maximum fermentation of galactose [36].

PTS 11 is a homolog for the PTS transporter in *L. acidophilus *(ORF 1012) which is induced in the presence of trehalose and is required for the utilization of trehalose [30]. In addition, LGAS_0533 is homologous to the phosphotrehalase (treC) characterized in *L. acidophilus *NCFM. While PTS 11 has an α-glucosidase near (treC), no predicted β-glucosidase is in the PTS 11 operon, suggesting that PTS 11 may not involved in β-glucoside uptake as annotated.

No PTS transporter that transports N-acetylglucosamine has been characterized in LAB. Based on our current knowledge, we can not predict which PTS transporter(s) can import N-acetylglucosamine.

We have identified several β-glucosides that are likely imported by PTS transporters including arbutin, salicin, gentiobiose, amygdalin and cellobiose. PTS 15 is the major cellobiose PTS transporter in *L. gasseri *ATCC 33323. Cellobiose PTS transporters have been identified that also transport other β-glucosides [37,38]. In addition, PTS 15 is a homolog to a PTS transporter in *Streptococcus mutans *that transports β-glucoside esculin [39]. PTS 5, 6, 8, 9, 15 and 19 all co-localize with potential β-glucosidases. Additional characterization is needed to identify which PTS transporters are involved in the utilization of β-glucosides.

## Conclusions

PTS transporters were confirmed to be largely important in the carbohydrate utilization potential of *L. gasseri *ATCC 33323. The PTS transporters were identified in various lactobacilli species using bioinformatic analysis. Comparative carbohydrate utilization assays were used to analyze the PTS content with carbohydrate utilization capability of three *L. gasseri *strains. The PTS carbohydrate specificity of transporters in *L. gasseri *ATCC 33323 was characterized by studying the transcript expression profiles in response to different carbohydrates. Lastly, the growth activity of selected PTS knockouts confirmed PTS transporter specificity predictions based on bioinformatics and transcript expression profiles. Our results confirm the importance of combining bioinformatics, transcript expression profiles and gene inactivation in identifying carbohydrate specificity of PTS transporters.

## Methods

### Bioinformatic Analysis

The genomes of *Lactobacillus acidophilus *NCFM, *L. brevis *ATCC 367, *L. casei *ATCC 334, *L. delbrueckii ssp. bulgaricus *ATCC 11842, *L. delbrueckii ssp. bulgaricus *ATCC BAA-365, *L. gasseri *ATCC 33323, *L. johnsonii *NCC 533, *L. plantarum *WCFS1, *L. reuteri *F275, *L. sakei ssp. sakei *23 K and *L. salivarius ssp. salivarius *UCC118 were analyzed using Concise Protein BLAST [40]. The PTS transporters of these strains were compared based on sequence similarity and function. PTS transporters were placed in the same cluster based on reciprocal best-hit blastP scores. Homologs were defined as PTS transporters that were in the same cluster.

The number of complete and incomplete PTS transporters present was determined for each species through bioinformatic analysis of the genomes. A complete PTS transporter was defined as having complete EIIA, EIIB and EIIC domains, which are required for PTS functionality [25]. An incomplete PTS transporter (also known as an orphan PTS) was defined as lacking in at least one of these domains. The sequential numbering of PTS transporters was based on their location in each respective genome. In order to identify non-PTS transporters with a PTS IIA domain, the conserved domain database was searched for PTS IIA domains [21,41].

### Bacterial Strains, Plasmids and Growth Conditions

The bacterial strains and plasmids used in this study are listed in Table 5. *L. gasseri *strains were grown at 37°C, in deMan, Rogosa, Sharpe (MRS) broth (Difco, Sparks, MD) or on MRS supplemented with 1.5% agar (Fisher, Fair Lawn, NJ). Agar plates were incubated anaerobically in a Coy anaerobic chamber (Grass Lake, MI) with a gas composition of 90% nitrogen, 5% hydrogen and 5% carbon dioxide. When necessary, erythromycin (Fisher) was added at a concentration of 2.5 μg/mL, and chloramphenicol (Fisher) was added at a concentration of 5 μg/mL. For the real-time PCR studies, *L. gasseri *ATCC 33323 was grown at 37°C in 10 mL semi-synthetic MRS medium supplemented with 1% carbohydrate (wt/vol) as previously described [42], except bromocresol purple was not used.

**Table 5:** Bacterial strains and plasmids

Strain or plasmid	Relevant characteristics	Source or reference
*L. gasseri*		
NCK334	ATCC 33323, human intestinal isolate	ATCC
MJM79	ATCC 33323 with pTRK669	This study
MJM75	ATCC 33323 EI::pMJM-1, EI^-^	This study
MJM99	ATCC 33323 PTS 15::pMJM-4, PTS 15^-^	This study
MJM100	ATCC 33323 PTS 20::pMJM-5, PTS 20^-^	This study
MJM101	ATCC 33323 PTS 21::pMJM-6, PTS 21^-^	This study
NCK100	ADH, human intestinal isolate	[43]
MJM55	ATCC 19992	ATCC
*E. coli*		
EC 1000	RepA^+ ^MC1000, Km^r^, carrying a single copy of the pWV01 *repA *gene in the *glgB *gene; host for pORI28-based plasmids	[44]
NCK1609	EC1000(pORI28)	[44]
NCK1391	EC1000(pTRK669)	[44]
MJM80	EC1000(pMJM-1)	This study
MJM103	EC1000(pMJM-4)	This study
MJM104	EC1000(pMJM-5)	This study
MJM105	EC1000(pMJM-6)	This study
Plasmids		
pORI28	Em^r^, *ori *(pWV01), replicates only with *repA *provided in *trans*	[44]
pTRK669	*ori *(pWV01), Cm^r^, provides *repA *in *trans*, temperature sensitive	[44]
pMJM-1	2.5 kb, pORI28 with 836-bp internal *L. gasseri *ATCC 33323 *EI *fragment	This study
pMJM-4	2.5 kb, pORI28 with 819-bp internal *L. gasseri *ATCC 33323 *PTS 15 *fragment	This study
pMJM-5	2.4 kb, pORI28 with 760-bp internal *L. gasseri *ATCC 33323 *PTS 20 *fragment	This study
pMJM-6	2.3 kb, pORI28 with 675-bp internal *L. gasseri *ATCC 33323 *PTS 21 *fragment	This study

*Escherichia coli *cells were grown at 37°C, in Luria-Bertani (LB) broth (Fisher) or on LB supplemented with 1.5% agar and grown anaerobically. When appropriate, kanamycin (Teknova, Hollister, CA) was added at a concentration of 40 μg/mL, erythromycin (Fisher) was added at a concentration of 150 μg/mL, and chloramphenicol (Fisher) was added at a concentration of 15 μg/mL.

### DNA Isolation, Manipulations and Transformations

Genomic DNA was isolated from *L. gasseri *ATCC 33323 using the Microbial DNA Isolation kit (MO BIO, Carlsbad, CA) according to the manufacturer's protocol. *E. coli *plasmid DNA was isolated using the QIAprep Spin Miniprep kit (QIAGEN).

DNA manipulations were carried out according to standard procedures. Restriction enzymes and T4 ligase were obtained from Invitrogen (Carlsbad, CA). When necessary, DNA fragments were isolated from agarose gels using the Zymoclean Gel DNA Recovery kit (Zymo Research, Orange, CA). PCR reactions were carried out according to standard procedures using EconoTaq polymerase from Lucigen (Middleton, WI). PCR primers were designed using Clone Manager 9 (Sci-Ed Software, Raleigh, NC) and purchased from IDT (Coralville, IA). For cloning purposes, restriction enzyme sites were added at the 5' end of the primers. PCR products were purified using the DNA Clean and Concentrator kit (Zymo Research).

Electrocompetent *L. gasseri *ATCC 33323 cells were prepared using 3.5× sucrose MgCl electroporation buffer as previously described [43]. To perform the electroporation, the Electroporator 2510 (Eppendorf, Westbury, NY) was used at a setting of 2.5 kV.

### PTS Content of *L. gasseri *Strains

Additional *L. gasseri *strains were analyzed for the presence of the fifteen complete PTS transporters found in *L. gasseri *ATCC 33323 using PCR. Genomic DNA isolated from *L. gasseri *33323, *L. gasseri *ADH and *L. gasseri *ATCC 19992 was used as templates in the PCR reactions. The same primers used for transcript analysis were used to determine the presence of homologous gene fragments (Table 6).

**Table 6:** Primer sequences used for transcript expression profiles and gene inactivation

Amplified Region	ORF	Primer Sequence	Product Size (bp)
PFK	881	5'-GTTATGGGTCGTGATGTG-3' (F)	85
		5'-AAGGCTCTTCTGGGATAAC-3' (R)	

PTS 1	118	5'-TTGGACGTGGCTTAGTTC-3' (F)	85
		5'-GCACCAGCTACTGTTAAACC-3' (R)	

PTS 3	149	5'-GATCAGGGCTAGTTGTTG-3' (F)	96
		5'-CGGCAGCTAAATAACCAC-3' (R)	

PTS 5	195	5'-GAAGCCTGCGTAAATAAGC-3' (F)	100
		5'-GTTGCCTGAACAAGTTCC-3' (R)	

PTS 6	343	5'-CGCAAATGGATACCATGAAAG-3' (F)	77
		5'-TCCAGTAGTGGTAATCATACG-3' (R)	

PTS 7	398	5'-GGAATGATGGGAAAGGGAATAG-3' (F)	99
		5'-TGCATGACCTGTTGGATAAG-3' (R)	

PTS 8	497	5'-AGCAACCCTATGACTACTACTC-3' (F)	92
		5'-AGCCATGGTAAGCACTTATC-3' (R)	

PTS 9	501	5'-CAACTTGTGCGAAGAATTTAAC-3' (F)	95
		5'-AATTTCAGCAGCTAAGATAACG-3' (R)	

PTS 10	515	5'-GCTCCAGCTTATGTCGTTAG-3' (F)	109
		5'-AGAAGCACCAGTTCGAATAG-3' (R)	

PTS 11	535	5'-GCCAGCCGTTTATGGTATC-3' (F)	82
		5'-AACAAGGCCACCAAATGC-3' (R)	

PTS 15	1669	5'-CTTCATTCCGATAGCATGTC-3' (F)	100
		5'-TGAAGGAGTTGCTGTTGAG-3' (R)	

PTS 17	1688	5'-TCCAGGTGTCTTGAAAGTAG-3' (F)	99
		5'-TCAGGGTGAGTGATAATGTC-3' (R)	

PTS 18	1726	5'-CCATACCACCTACCATAAACAG-3' (F)	91
		5'-GCTTACGTCTTTGCTTCAG-3' (R)	

PTS 19	1755	5'-CAATCATTGCACCATACATAGG-3' (F)	78
		5'-ATTTAGGCGGAATTACAGAAC-3' (R)	

PTS 20	1778	5'-GCGCTTACCGTATACAAAGG-3' (F)	89
		5'-TGGTGCCAAAGAGTATTCC-3' (R)	

PTS 21	1794	5'-GCAGGAATGGCAAGTAATAAAC-3' (F)	86
		5'-GCTATTGATCGTTGGCAAATG-3' (R)	

AF_1360Bam	1360	5'-ATGCGGATCC-CGGCAGCCATAGTATATTG-3' (F)	836
AF_1360Nco		5'-ATGCCCATGG-TCATGGCTCGTTCATTAG-3' (R)	

AF_ori+	--	5'-GATAATGAACTGTGCTGATTAC-3' (F)	1071
AF_EI+		5'-TGGGTTATATGGTTGGTAAAG-3' (R)	

AF_ori-	--	5'-TTCAATCGCCAACGAATC-3' (F)	1020
AF_EI-		5'-AGTGATACAGCTCAACTTAAC-3' (R)	

MM_1669Bam	1669	5'-AGTCGGATCCTGCAGCAGGTATGATTAAAG-3' (F)	819
MM_1669Nco		5'-AGTCCCATGGAATAGCTGGTTCAGTAACAC-3' (R)	

AF_ori+	--	5'-GATAATGAACTGTGCTGATTAC-3' (F)	999
MM_PTS15+		5'-TGCTGATGACGATTTAGATG-3' (R)	

AF ori-	--	5'-TTCAATCGCCAACGAATC-3' (F)	1039
MM_PTS15-		5'-GCTGCAATACAACTTAAGAC-3' (R)	

MM_1778Bam	1778	5'-AGTCGGATCCCAGGTTTGTTTGGAGCAAAG' (F)	760
MM_1778Nco		5'-AGTCCCATGGTGCTGGTTCAGTAATACCAAG-3' (R)	

AF_ori+	--	5'-GATAATGAACTGTGCTGATTAC-3' (F)	894
MM_PTS20+		5'-CTTAGTAGCTGGTGGTTTG-3' (R)	

AF ori-	--	5'-TTCAATCGCCAACGAATC-3' (F)	990
MM_PTS20-		5'-TACACCTGCACCAATTAAAG-3' (R)	

MM_1795Bam	1795	5'-AGTCGGATCCAAGGTCCTGATGATATTAGAG' (F)	675
MM_1795Nco		5'-AGTCCCATGGATAGCTTTAAGCGCATCTTC-3' (R)	

AF_ori+	--	5'-GATAATGAACTGTGCTGATTAC-3' (F)	854
MM_PTS21+		5'-TAGTCACGGTGGCTTTG-3' (R)	

AF ori-	--	5'-TTCAATCGCCAACGAATC-3' (F)	895
MM_PTS21-		5'-CACTGTAAGCCATGGAAC-3' (R)	

### *EI *Gene Inactivation

The inactivation of *EI *was performed by targeted insertion of an erythromycin resistant, non-replicative vector pMJM-1 by homologous recombination using a previously established method [44]. Plasmid pMJM-1 was designed to disrupt the *L. gasseri *ATCC 33323 *EI *gene, encoding for enzyme I of the PTS system. The primers AF_1360Bam and AF_1360Nco (Table 6) were used to amplify an 836 bp internal region of *EI *from *L. gasseri*. This fragment was cloned via the *Bam*HI/*Nco*I sites into pORI28, an Ori^+^, RepA^- ^integration plasmid. Plasmid pMJM-1 was introduced into *L. gasseri *containing pTRK669 (MJM79) by electroporation. RepA function was provided by the helper plasmid pTRK669, which is stable at 37°C but not at 43°C. Transformants carrying both plasmids were transferred five times (overnight transfers) and allowed to grow at 43°C in MRS broth supplemented with erythromycin (2.5 μg/mL) to avoid the insertion of multiple copies of the vector.

The occurrence of single cross-over events was verified by PCR amplification of the junction fragments from chromosomal DNA of Em^r^-Cm^s ^colonies. *EI *specific external primers and specific internal primers for the Em gene in the vector were used to confirm successful insertion of pMJM-1 into the *EI *gene. The 5' junction fragment, demonstrating integration in the *EI *gene (the primers AF_ori+ and AF_EI+ were used - Table 6) had an expected size of 1071 bp. The 3' junction fragment, demonstrating integration in the *EI *gene (the primers of AF_ori- and AF_EI- were used - Table 6) had an expected size 1020 bp. MJM75 had the expected junction fragments and is an *EI *knockout.

### PTS 15, 20 and 21 Gene Inactivation

The inactivation of PTS 15, 20 and 21 followed the same general outline as the *EI *gene inactivation. The non-replicative vectors pMJM-4, pMJM-5 and pMJM-6 were used to inactivate PTS 15, 20, and 21, respectively (Table 5). The amplified PTS 15 (LGAS_1669), 20 (LGAS_1778) and 21 (LGAS_1795) internal regions were 819 bp, 760 bp and 675 bp, respectively. The junction fragments for successful pMJM-4 integration were 999 bp and 1039 bp. The junction fragments for successful pMJM-5 integration were 894 bp and 990 bp. The junction fragments for successful pMJM-6 integration were 854 bp and 895 bp. MJM99, MJM100 and MJM101 had the expected junction fragments and are PTS 15, PTS 20 and PTS 21 knockouts, respectively.

### Carbohydrate Utilization Analysis

Strains were analyzed for their ability to utilize carbohydrates with the API 50 carbohydrate utilization assay (bioMérieux, Durham, NC) according to the manufacturer's protocol. Strains analyzed are as follows: *L. gasseri *ATCC 33323, *L. gasseri *ATCC 33323 *EI*::MJM75, *L. gasseri *ADH, *L. gasseri *ATCC 19992, *L. gasseri *ATCC 33323 *PTS 15*::MJM99, *L. gasseri *ATCC 33323 *PTS 20*::MJM100, and *L. gasseri *ATCC 33323 *PTS21*::MJM101.

For the growth experiments, *L. gasseri *strains were first grown in MRS. After two passes, the strains were inoculated into semi-synthetic MRS medium supplemented with 1% carbohydrate (wt/vol). The growth curve was generated using the protocol described by Barboza et al. [45]. Briefly, 100 μl of inoculated media was placed into a sterile 96-well plate and then topped with 40 μL of mineral oil. The plate was incubated at 37°C in an anaerobic chamber with OD_600 nm _readings taken every 30 minutes.

### RNA Isolation and Analysis

RNA was isolated from *L. gasseri *ATCC 33323 using the Microbial RNA Isolation kit (MO BIO) according to the manufacturer's protocol. Semi-synthetic MRS was used to analyze PTS gene expression in response to various carbohydrates. The carbohydrates added to the medium were glucose (Fisher), mannose (Acros Organics, NJ), fructose (Sigma-Aldrich, St. Louis, MO), sucrose (Fisher), or cellobiose (Acros Organics). 0.1% of overnight culture was transferred 6 times before isolation of RNA. The final transfer of *L. gasseri *was grown to an OD_595 nm _of 0.6 in order to obtain mid-log phase cells [42]. 1.5 mL of culture was collected by centrifugation at 10,000 × *g *at room temperature. RNA was isolated from the cells using the UltraClean Microbial RNA Isolation Kit according to manufacturer's protocol (MO BIO). To eliminate contaminating DNA, 100 ng/μL of RNA was treated with TURBO DNA-*free *according to the supplier's instructions in a 50 μL reaction volume (Ambion, Austin, TX).

Two-step real-time PCR was performed to carry out the relative quantification of the fifteen complete PTS transporters from the five different conditions (glucose, mannose, fructose, sucrose and cellobiose). The reverse transcription step was performed using the iScript cDNA sythesis kit to convert the RNA samples to cDNA according to the manufacturer's protocol (BioRad, Hercules, CA). Typically, 0.8 μg of RNA was converted to cDNA in a 20 μL reaction volume. The iScript PCR reaction conditions used are as follows. The reaction mixture was held at 25°C for 5 minutes, 42°C for 30 minutes, heated to 85°C for 5 minutes, and stored at 4°C (Biorad, Hercules, CA). The quantification step of real-time PCR was performed using iTaq SYBR Green Mastermix with ROX (Biorad). Primers were designed for the 15 complete PTS transporters in *L. gasseri *ATCC 33323 using Clone Manager 9 (Sci-Ed Software) and are shown in Table 6. The IIC component of each of the fifteen complete PTS transporters was targeted for primer design. Primers used in the real-time experiments were synthesized by Invitrogen.

Relative quantification of the transcription profiles of the fifteen complete PTS transporters in *L. gasseri *ATCC 33323 was performed using the 7300 Real-time PCR System (Applied Biosystems, Foster City, CA). Typically, 5 μL of cDNA (0.8 μg) was added to the reaction mixture consisting of 12.5 μL iTaq SYBR Green Mastermix with ROX (BioRad), 1 μL of the forward primer (5 μM), 1 μL of the reverse primer (5 μM), and 5.5 μL DEPC water (MO BIO). The reaction mixture was held at 95°C for 2 minutes, 95°C for 15 seconds and 60°C for one minute (repeated 35 times), 95°C for 15 seconds, 60°C for 1 minute, 95°C for 15 seconds, and 60°C for 15 seconds. Relative fold changes were reported by using a phosphofructokinase (PFK) gene in *L. gasseri *(Table 6 - PFK primer sequences) that was previously shown in *L. plantarum *WCFS1 to exhibit qualities of an acceptable internal standard [46]. The ^ΔΔ^Ct method [47] was used to calculate the relative fold change of the PTS systems using fructose as the calibrator. Reported relative fold changes are the average of three independent experiments +/- the standard deviation.

## Authors' contributions

ALF performed the majority of the experiments, participated in bioinformatic analysis, study design, and in crafting of the manuscript. TT performed the growth experiments. MJM created MJM99, MJM100, and MJM101, conceived the study, participated in the design, coordination, bioinformatic analysis, and crafting of the manuscript.
